# Dietary, Lifestyle and Socio-Economic Correlates of Overweight, Obesity and Central Adiposity in Lebanese Children and Adolescents

**DOI:** 10.3390/nu6031038

**Published:** 2014-03-10

**Authors:** Lara Nasreddine, Farah Naja, Christelle Akl, Marie Claire Chamieh, Sabine Karam, Abla-Mehio Sibai, Nahla Hwalla

**Affiliations:** 1 Department of Nutrition and Food Sciences, Faculty of Agricultural and Food Sciences, American University of Beirut, P.O. Box 11-0236, Riad El Solh, Beirut 1107-2020, Lebanon; E-Mails: ln10@aub.edu.lb (L.N.); fn14@aub.edu.lb (F.N.); cristell@gmail.com (C.A.); mc31@aub.edu.lb (M.C.C.); sek04@aub.edu.lb (S.K.); 2 Department of Epidemiology and Population Health, Faculty of Health Sciences, American University of Beirut, P.O. Box 11-0236, Riad El Solh, Beirut 1107-2020, Lebanon

**Keywords:** paediatric, obesity, abdominal adiposity, prevalence, correlates, diet, Lebanon, Eastern Mediterranean region

## Abstract

The Eastern Mediterranean region is characterized by one of the highest burdens of paediatric obesity worldwide. This study aims at examining dietary, lifestyle, and socio-economic correlates of overweight, obesity, and abdominal adiposity amongst children and adolescents in Lebanon, a country of the Eastern Mediterranean basin. A nationally representative cross-sectional survey was conducted on 6–19-year-old subjects (*n* = 868). Socio-demographic, lifestyle, dietary, and anthropometric data (weight, height, waist circumference) were collected. Overweight and obesity were defined based on BMI *z*-scores. Elevated waist circumference (WC) and elevated waist to height ratio (WHtR) were used as indices of abdominal obesity. Of the study sample, 34.8% were overweight, 13.2% were obese, 14.0% had elevated WC, and 21.3% had elevated WHtR. Multivariate logistic regression analyses showed that male gender, maternal employment, residence in the capital Beirut, sedentarity, and higher consumption of fast food and sugar sweetened beverages were associated with increased risk of obesity, overweight, and abdominal adiposity, while regular breakfast consumption, higher intakes of milk/dairies and added fats/oils were amongst the factors associated with decreased risk. The study’s findings call for culture-specific intervention strategies for the promotion of physical activity, healthy lifestyle, and dietary practices amongst Lebanese children and adolescents.

## 1. Introduction

The Eastern Mediterranean region is characterized by one of the highest burdens of overweight and obesity worldwide [1]. Of more concern is the high level of childhood obesity in countries of the region, with approximately 10% of school-aged children being obese, an estimate that is projected to follow an escalating secular trend [2]. Paediatric obesity is associated with both immediate and longer-term risks to health [3]. Among the immediate risks are metabolic abnormalities including increased blood cholesterol, triglycerides and glucose levels, insulin resistance, metabolic syndrome, and hypertension [3,4,5]. Childhood obesity is also a strong risk factor for adult obesity and its consequences including type 2 diabetes, cardiovascular diseases (CVDs), and certain types of cancer, in addition to psychological disturbances, such as low self-esteem and depression [6,7].

Obesity-related comorbidities were found to be more closely associated with abdominal adiposity and visceral fat depots than with the amount of total body fat [8]. Consequently, the use of body fat distribution indices has been increasingly recommended, and particularly the use of waist circumference (WC) and waist to height ratio (WHtR). These simple and non-invasive indices were shown to correlate with visceral fat in children and to predict risk for obesity-related comorbidities beyond that predicted by Body Mass Index (BMI) alone [8,9,10,11,12,13,14]. Being a relatively age-independent measure, the use of WHtR for assessing central fatness in children has been recommended in paediatric primary care practice, as well as epidemiological studies [14,15,16]. In a cohort of almost 1500 Caucasian children aged 5 to 15 years, both WC and WtHR were able to identify children with the highest metabolic and cardiovascular risks among those who were overweight [13]. An extensive review by Huxley *et al.* (2010) concluded that measures of abdominal obesity including WC and WHtR, may be better than BMI in predicting CVD risk, although combining BMI with these measures may improve their discriminatory capability [17].

The high disease burden of childhood obesity highlights the need for rigorous investigations of its determinants, context-specific patterns and associated factors. Most of the studies investigating obesity correlates in youth have been conducted in high-income countries and, as such, findings may not be applicable to low and middle-income countries. Among the latter, the Middle-East has been largely under-represented, although the region has one of the highest rates of childhood obesity [2]. The present study aims at examining the prevalence and correlates of overweight, obesity and abdominal adiposity in a nationally representative sample of children and adolescents, aged six years and above, in Lebanon. Gaining greater insight into factors that are associated with paediatric obesity could catalyze the development of effective interventions and policies aiming at curbing the obesity epidemic in Lebanon, orient further studies, and assist policy makers in implementing successful, culture specific childhood obesity prevention strategies in the region.

## 2. Materials and Methods

### 2.1. Study Design and Subjects

Data for the present study is drawn from a national cross-sectional survey that was conducted in 2009, in Lebanon, on subjects aged six years and above. The study sample was based on the sampling frame provided by the National Survey of Household Living Conditions, which was conducted by the Ministry of Social Affairs/Central Administration of Statistics in collaboration with United Nations Development Programme (UNDP) and which covered primary residences across the Lebanese territory [18]. Sample size calculation for the study was performed based on previously estimated prevalence rates for the main outcome of interest [19]. As such, a minimum of 751 participants were needed to estimate a prevalence of obesity of 4.8% in children and adolescents [19], allowing a power of 80% and a margin of error of 1.5% at 95% confidence interval (CI). Recruitment efforts targeted a sample with an age, sex and district distribution proportionate to that of the Lebanese population [18].

Lebanon is divided into six administrative regions referred to as “governorates”, which cover the totality of the country. Except for the governorate of Beirut, which is considered purely urban, the other governorates are essentially composed of rural regions inter-mixed with urban cities. In this study, the sample was drawn from randomly selected households, based on stratified cluster sampling: the strata were the Lebanese governorates, the clusters were selected further at the level of districts, urban and rural areas, and the housing units constituted the primary sampling units in the different districts of Lebanon. One adult from each household and one child/adolescent from every other household were selected from the household roster. Field-work was carried out between May 2008 and August 2009. The final sample consisted of 3636 subjects, including 939 children and adolescents aged 6 years and above [20]. Refusal rate at the household level was estimated at 10.7%, with the main reasons for refusal to participate in the survey being lack of time or disinterest in the study. The design and conduct of the survey was approved by the Institutional Review Board of the American University of Beirut, and informed consent from adults/parents and informed assent from children and adolescents were obtained prior to enrolment in the studies.

Socio-demographic and lifestyle data were collected from study participants using a multi-component questionnaire that was developed for the purpose of this study. Data collection was performed by trained nutritionists in the household setting through face to face interviews which lasted for approximately one hour. Quality control measures including training, pre-testing of the study instruments, equipment, and data collection procedure and field monitoring of data collection, were applied. Household and parental data were collected from the adult participant (mother or father) using a multicomponent questionnaire covering information on demographic, socioeconomic and lifestyle characteristics, in addition to medical history and health seeking behavior. Data pertinent to the child/adolescent were collected using a child-specific questionnaire which enquired about sex, age, medical history, meal pattern, eating habits, dietary intake, physical activity, and sedentary time. For children aged less than 11 years old, data was obtained by proxy (typically the mother), while the interview was conducted directly with subjects aged 11 years and above.

### 2.2. Anthropometric Measurements

Anthropometric measurements were taken using standardized protocols [21] and calibrated equipment. Height and body weight were measured according to standard procedures, using a portable stadiometer (Holtain, Crymych, UK) and a Secacalibrated electronic weighing scale (Hamburg, Germany), respectively. Subjects were weighed to the nearest 0.1 kg in light indoor clothing and with bare feet or stockings. Height was measured without shoes and recorded to the nearest 0.5 cm. A calibrated plastic measuring tape was used to measure waist circumference at the level of the umbilicus to the nearest 0.1 cm, with the subject standing and after normal expiration. Anthropometric measurements were taken and recorded by trained nutritionists who were working in teams of two, the examiner and the recorder. All measurements were taken twice and the average of the 2 values was adopted.

### 2.3. Definitions of Overweight and Obesity

Body mass index (BMI) was calculated as the ratio of weight (kilograms) to the square of height (meters). Overweight and obesity were defined based on sex and age specific +1 and +2 BMI *z*-scores, respectively, according to the WHO new growth standards [22]. The WHO AnthroPlus software (WHO, Geneva, Switzerland) was used to calculate BMI *z*-score for each specific age and sex. To allow for comparisons with studies conducted in other countries, prevalence rates of overweight and obesity were also determined using the International Obesity Taskforce (IOTF) [23] and the US Centers for Disease Control and Prevention (CDC) 2000 criteria [24].

Elevated WC was defined based on the International Diabetes Federation (IDF) criteria [25], which recommend the use of:

- Adult cut-off values for subjects aged 16–19 years (WC > 94 cm for males and >80 cm for females).
- Cut-off value of WC ≥ 90th percentile for sex and age (or adult cut-offs if lower) for subjects aged 6 to 15 years old. As national WC percentiles are lacking in Lebanon, the WC percentiles for children and adolescents as developed by Fernandez *et al.* (2004) were used [26].

The WHtR index for abdominal obesity was calculated by dividing WC by height, both measured in centimetres [13]. The suggested cut-off point of ≥0.5 was used to identify children with elevated WHtR [13,14].

### 2.4. Dietary Intake and Physical Activity Assessment

Dietary intake was assessed using the multiple pass 24-h recall approach. Interviewers followed the 5 steps of the USDA multiple pass 24-h recall, which included (1) the quick list; (2) the forgotten foods list; (3) time and occasion at which foods were consumed; (4) the detail cycle; and (5) the final probe review [27]. To assist subjects in assessing the portion/amount of food consumed, quantification tools, such as household measures and graduated food models, were used. 24-h recall data were converted to energy and nutrient intake using the Nutritionist IV software through a hand-coding procedure (*N*-squared Computing Nutritionist IV. Silverton, OR: *N*-squared Computing; 1995). The Nutritionist IV food database was expanded by adding analyses of traditional Lebanese foods and recipes.

Information on the weekly frequency of physical activity outside the school setting was assessed by means of a questionnaire that was developed for this study. Examples of activities proposed by the questionnaire included moderate intensity activities such as playground activities, brisk walking, dancing, bicycle riding, as well as higher intensity activities, such as ball games, jumping rope, active games involving running and chasing, and swimming. Based on the weekly frequency, individuals were classified into three levels of physical activity: Low (Never); Moderate (1–2 times/week) and High (>2 times/week).

### 2.5. Statistical Analysis

Descriptive statistics were performed and expressed as means and standard error (SE) for continuous variables (dietary variables) or as number of subjects and percentages for nominal variables (demographic, socio-economic, physical activity, meal pattern, and lifestyle variables). Crowding index was calculated as the total number of co-residents per household divided by the total number of rooms, excluding the kitchen and bathrooms. Prevalence of overweight (including obesity), obesity, elevated WC and elevated WHtR, expressed as percentage with 95% confidence interval (CI), were computed by gender and age groups (6–11-year-old children and 12–19-year-old adolescents). Independent *t*-test and chi-squared test were used to evaluate the differences between continuous and categorical variables, respectively.

Multivariate logistic regression analysis was carried out to examine the association of overweight, obesity, elevated WC and elevated WHtR as the dependent variables with baseline socio-demographic, lifestyle, and dietary characteristics as covariates. The associations between dependent and independent variables were analyzed according to two age groups: Children (6–11 years) and adolescents (12–19 years). All statistical calculations were carried out using the Statistical Analysis Package for Social Sciences, version 18.0 (SPSS Inc., Chicago, IL, USA). Statistical significance was defined as *p*-value <0.05.

## 3. Results

### 3.1. Study Sample

For the purpose of this study, subjects for whom dietary data were missing or incomplete were excluded (out of 939 subjects, 71 were excluded). Accordingly, the final study sample consisted of 868 subjects (439 boys and 429 girls), with a mean age of 13.06 years (±3.91) and a median age of 12.85 years. Of the study participants, 42.6% were 6–11 years old and 57.4% were 12–19 years old. The male to female ratio was of 1.02 with 50.6% boys and 49.4% females.

The proportion of parents who had attained high school level education and above was of 38.6% for fathers and 46.3% for mothers with no significant differences between age groups (Table 1). A significantly higher proportion of working mothers was reported amongst 12–19-year-old adolescents (29.6%) compared to children (19.4%). Parental obesity (mother or father) was reported amongst 31% of the study population with no significant differences between age groups. The majority of subjects (81.7%) had a crowding index ≥1 persons/room (Table 1).

The proportions of subjects reporting a daily consumption of breakfast was significantly higher amongst children compared to adolescents (86.4% *vs.* 69.5% in adolescents) while a significantly higher proportion of adolescents reported eating outside home more than once per week (58.4% in adolescents compared to 44.4% in children) (Table 1). Similarly, sedentary time was significantly higher amongst 12–19-year-old adolescents compared to children (10.09 ± 2.94 *vs.* 8.72 ± 2.77 h/day) while the proportion of subjects reporting high physical activity was significantly higher in children compared to adolescents (63.9% *vs.* 33.5%).

Mean weight (35.01 ± 12.55 in children *vs.* 60.77 ± 15.61 kg in adolescents), mean height (135.63 ± 12.26 *vs.* 164.17 ± 10.07 cm), mean BMI (18.53 ± 3.99 *vs.* 22.32 ± 4.37 kg/m^2^) and mean WC (63.77 ± 10.75 *vs.* 74.93 ± 10.97 cm) were all significantly higher in 12–19-year-old adolescents compared to children aged 6–11 years (Table 1).

**Table 1:** Socio-demographic, lifestyle and anthropometric characteristics of the study sample by age group, Lebanon (*n* = 868).

Variables	Age Group (years)		Total ^(1)^	*p*-Value ^(2)^
	6–11 (*n* = 370)	12–19 (*n* = 498)	(*n* = 868)	
**Socio-Demographic characteristics *n* (%)**				
**Gender**				
Male	191 (51.6)	248 (49.8)	439 (50.6)	0.595
Female	179 (48.4)	250 (50.2)	429 (49.4)	
**Governorates**				
Capital (Beirut)	26 (7.0)	36 (7.2)	62 (7.1)	0.909
Other governorates	344 (93.0)	462 (92.8)	806 (92.9)	
**Father’s Education**				
Primary or less	105 (28.8)	156 (31.7)	261 (30.5)	0.571
Intermediate	112 (30.8)	153 (31.1)	265 (31.0)	
High school and above	147 (40.4)	183 (37.2)	330 (38.6)	
**Mother’s Education**				
Primary or less	76 (23.0)	112 (26.3)	188 (24.9)	0.587
Intermediate	97 (29.4)	121 (28.4)	218 (28.8)	
High school and above	157 (47.6)	193 (45.3)	350 (46.3)	
**Mother’s working status**				
Not working	291 (80.6)	350 (70.4)	641 (74.7)	0.001
Working	70 (19.4)	147 (29.6)	217 (25.3)	
**Parental Obesity ^(3)^**				
No	218 (71.5)	171 (66.0)	389 (69.0)	0.163
Yes	87 (28.5)	88 (34.0)	175 (31.0)	
**Crowding Index**				
<1 person/room	66 (18.0)	92 (18.5)	158 (18.3)	0.858
≥1 person/room	300 (82.0)	405 (81.5)	705 (81.7)	
**Lifestyle characteristics *n* (%)**				
**Breakfast consumption (per week)**				
Never	11 (3.0)	36 (7.2)	47 (5.4)	<0.001
Sometimes	39 (10.6)	116 (23.3)	155 (17.9)	
Daily	319 (86.4)	346 (69.5)	665 (76.7)	
**Frequency of eating outside home (per week)**				
≤1 time	205 (55.6)	207 (41.6)	412 (47.5)	<0.001
>1 time	164 (44.4)	291 (58.4)	455 (52.5)	
**Physical Activity ^(4)^**				
Low	80 (21.7)	193 (41.2)	273 (32.6)	<0.001
Moderate	53 (14.4)	119 (25.4)	172 (20.5)	
High	235 (63.9)	157 (33.5)	392 (46.8)	
**Sedentary time** (h/day) Mean ± SD	8.72 ± 2.77	10.09 ± 2.94	9.51 ± 2.95	<0.001
**Anthropometric characteristics**				
**Weight (kg)**				
Mean ± SD	35.01 ± 12.55	60.77 ± 15.61	49.81 ± 19.21	<0.001
10th percentile	22.15	42.67	25.88	
50th percentile	32.45	59	48.75	
90th percentile	50.81	80.81	74.52	
**Height (cm)**				
Mean ± SD	135.63 ± 12.26	164.17 ± 10.07	152.02 ± 17.93	<0.001
10th percentile	119.75	152	126	
50th percentile	136	164	154	
90th percentile	152	177.5	174.05	
**BMI (kg/m^2^)**				<0.001
Mean ± SD	18.53 ± 3.99	22.32 ± 4.37	20.71 ± 4.61	
10th percentile	14.65	17.47	15.6	
50th percentile	17.58	21.58	20.08	
90th percentile	23.56	28.17	26.8	
**WC (cm)**				
Mean ± SD	63.77 ± 10.75	74.93 ± 10.97	70.18 ± 12.19	<0.001
10th percentile	52.75	62.73	56	
50th percentile	61.5	73.2	69	
90th percentile	78.5	91	86.05	
**WHtR**				
Mean ± SD	0.47 ± 0.06	0.46 ± 0.06	0.46 ± 0.06	0.002
10th percentile	0.41	0.39	0.4	
50th percentile	0.46	0.44	0.45	
90th percentile	0.55	0.54	0.55	

^(1)^ Lack of corresponding sum of frequencies with total sample size is due to missing data; ^(2)^ Differences between age groups were examined using *t*-test and chi-square test for continuous and categorical variables, respectively; ^(3)^ Total number of parents with anthropometric data was equal to 564; ^(4)^ The three categories of physical activity (Low, Moderate, High) refer to the frequency of physical activity outside the school setting (Never; 1–2 times/week; >2 times/week, respectively).

### 3.2. Prevalence of Overweight, Obesity and Abdominal Obesity

Taking both genders, 40.2% of 6–11-year-olds and 30.8% of 12–19-year-olds were found to be overweight (BMI *z* score >+1), while 17.1% and 10.3% were found to be obese (BMI *z* score >+2), respectively (Table 2). Gender-based differences were noted amongst 12–19-year-olds, with the prevalence of overweight (37.9% in boys *vs.* 23.7% in girls) and obesity (16.1% in boys *vs.* 4.4% in girls) being significantly higher in boys compared to girls. Similar gender-based differentials were noted in the total sample of 6–19-year-old subjects.

Based on WC as an indicator of central fatness, abdominal obesity was observed in 13.8% of 6–11-year-olds and 14.1% of 12–19-year-olds, with no significant differences between genders (Table 2). Elevated WHtR was observed in 22% and 20.9% of children and adolescents, respectively, with gender-based differences being observed amongst 12–19-year-old subjects (26.2% in boys *vs.* 15.6% in girls; *p* < 0.05). Similar gender-based differentials were noted in the prevalence of elevated WHtR in the total sample of 6–19-year-old subjects (Table 2).

**Figure 1:**
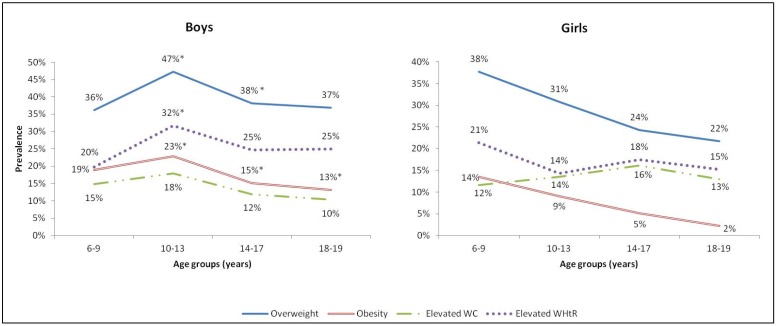
Prevalence of overweight, obesity, elevated WC and elevated WHtR amongst Lebanese children and adolescents (*n* = 868) by age and gender (* Significant difference by gender *p* < 0.05).

As shown in Figure 1, the prevalence of overweight, obesity, elevated WHtR and elevated WC amongst boys reached the highest rates at the age of 10–13 years (47%, 23%, 32% and 18%, respectively), while declining afterwards (Figure 1). Amongst girls, the prevalence of overweight and obesity was the highest at 6–9 years (38% and 14%, respectively) and followed a consistent declining trajectory with age. The prevalence of elevated WC reached its highest in girls aged between 14 and17 years (16%) and declined afterwards (Figure 1).

**Table 2:** Prevalence of overweight, obesity and abdominal adiposity amongst Lebanese children and adolescents (*n* = 868) by gender and by age group.

Variables	Age Groups (years)						Total		
	6–11			12–19			6–19		
	*n*	%	(95% CI)	*n*	%	(95% CI)	*n*	%	(95% CI)
**Male**									
Overweight ^(1)^	81	42.4	(36–50)	94	37.9 ^a^	(32–44)	175	39.9 ^a^	(35–44)
Obesity ^(1)^	39	20.4	(15–27)	40	16.1 ^b^	(12–21)	79	18.0 ^b^	(15–22)
Elevated WC ^(2)^	30	15.7	(11–22)	32	12.9	(9–18)	62	14.1	(11–18)
Elevated WHtR ^(3)^	46	24.1	(19–31)	65	26.2 ^c^	(21–32)	111	25.3 ^c^	(21–30)
**Female**									
Overweight ^(1)^	67	37.9	(31–45)	59	23.7 ^a^	(19–29)	126	29.6 ^a^	(25–34)
Obesity ^(1>)^	24	13.6	(9–19)	11	4.4 ^b^	(2–8)	35	8.2 ^b^	(6–11)
Elevated WC ^(2)^	21	11.8	(8–17)	38	15.2	(11–20)	59	13.8	(11–17)
Elevated WHtR ^(3)^	35	19.7	(14–26)	39	15.6 ^c^	(12–21)	74	17.3 ^c^	(14–21)
**Both Genders**									
Overweight ^(1)^	148	40.2	(35–45)	153	30.8	(27–35)	301	34.8	(32–38)
Obesity ^(1)^	63	17.1	(14–21)	51	10.3	(8–13)	114	13.2	(11–16)
Elevated WC ^(2)^	51	13.8	(10–17)	70	14.1	(11–17)	121	14.0	(11–16)
Elevated WHtR ^(3)^	81	22.0	(18–27)	104	20.9	(17–24)	185	21.3	(18–24)

WC: Waist Circumference; WHtR: Waist to Height ratio; ^(1)^ Overweight and obesity defined based on sex and age specific +1 and +2 BMI *z*-scores, respectively [22]; ^(2)^ For subjects aged 6–15 years, abdominal obesity: WC > 90th percentile [26] or adult cut-off value if lower [25]; For subjects aged 16–19 years, abdominal obesity: WC > 94 cm for males and >80 cm for females [25]; ^(3)^ Elevated WHtR defined as WHtR > 0.5 [13]; ^a,b,c^ Within each age group, values with the same superscripts are significantly different by gender at *p* < 0.05 (Using Chi-square test).

### 3.3. Dietary Intake

As shown in Table 3, average energy intake (2255.85 *vs.* 1736.48 kcal/day) and percent contribution of fast food (17.27% *vs.* 11.35%) and legumes and nuts (3.28% *vs.* 2.05%) to daily energy intake were significantly higher among 12–19-year-old adolescents compared to 6–11-year-old children. On the other hand, the percent contribution of milk and dairies (8.90% *vs.* 6.47%) and breads and cereals (36.92% *vs.* 32.67%) were significantly higher in 6–11-year-old children compared to adolescents. No significant differences in macronutrient intake were observed between age groups.

**Table 3:** Energy, macronutrient and food group intake amongst Lebanese children and adolescents according to age group (*n* = 868).

Dietary Variables	Age Group (years)				Total	
	6–11 (*n* = 370)		12–19 (*n* = 498)		6–19 (*n* = 868)	
Energy (kcal ± SE )	1736.48 ± 36.12 ^a^		2255.85 ± 51.60 ^a^		2033.70 ± 34.47	
	**Mean % Daily Energy Intake ± SE**					
Carbohydrates	52.01 ± 0.51		51.05 ± 0.48		51.46 ± 0.35	
Protein	13.11 ± 0.18		13.53 ± 0.23		13.35 ± 0.15	
Fat	35.86 ± 0.47		36.24 ± 0.43		36.058 ± 0.32	
Breads and Cereals	36.92	±0.89 ^b^	32.67	±0.77 ^b^	34.49	±0.58
Milk and Dairies	8.90	±0.52 ^c^	6.47	±0.37 ^c^	7.51	±0.31
Meat and Equivalent	10.22	±0.57	10.15	±0.57	10.18	±0.40
Legumes and Nuts	2.05	±0.34 ^d^	3.28	±0.38 ^d^	2.76	±0.27
Fruits and Vegetables	5.35	±0.33	5.42	±0.33	5.39	±0.24
Added Fats and Oils	7.58	±0.47	8.42	±0.42	8.06	±0.31
Fast Food	11.35	±0.69 ^e^	17.27	±0.92 ^e^	14.74	±0.61
Sugar and Sweets	10.81	±0.63	9.65	±0.60	10.15	±0.44
Sugar Sweetened Beverages	6.52	±0.45	6.45	±0.32	6.48	±0.26

^a,b,c,d,e^ Values with the same superscripts are significantly different by age group at *p* < 0.05 (Using *t*-test).

### 3.4. Factors Associated with Overweigh, Obesity and Abdominal Obesity

Amongst 6–11-year-old children, results of the multivariate regression analysis showed that, as compared to subjects living in the capital Beirut, those residing in other governorates had significantly lower odds of being overweight (OR = 0.32; 95% CI: 0.1–0.98), obese (OR = 0.21; 95% CI: 0.06–0.71), and of having elevated WC (OR = 0.16; 95% CI: 0.04–0.56) (Table 4). Higher maternal education was associated with significantly higher odds of overweight (OR = 2.45; 95% CI: 1.13–5.31), while higher paternal education was associated with lower odds of obesity in this age group (OR = 0.32; 95% CI: 0.11–0.91). Maternal employment was shown to be associated with significantly higher odds of obesity (OR = 2.6; 95% CI: 1.18–5.70) and elevated WHtR (OR = 2.27; 95% CI: 1.19–4.33). In contrast, daily breakfast consumption was associated with significantly lower odds of overweight (OR = 0.2; 95% CI: 0.05–0.84) and obesity (OR = 0.07; 95% CI: 0.01–0.30); higher intakes of milk & dairies were associated with lower odds of elevated WC (OR = 0.35; 95% CI: 0.13–0.91), and higher intakes of added fats/oils were associated with lower odds of obesity (OR = 0.30; 95% CI: 0.12–0.73), elevated WHtR (OR = 0.42; 95% CI: 0.20–0.88), and elevated WC (OR = 0.32; 95% CI: 0.12–0.88). High consumption of fast food was associated with a threefold increase in the risk of overweight in this age group (OR = 3.24; 95% CI: 1.21–8.69) (Table 4).

**Table 4:** Associations of socio-demographic, lifestyle and dietary factors with overweight, obesity, elevated waist to height ratio (WHtR), and elevated waist circumference (WC) in Lebanese 6–11-year-old children (*n* = 868).

Variables	6–11 Years			
	(*n* = 370)			
	Overweight ^1^	Obesity ^1^	Elevated WHtR	Elevated WC
	Odds Ratio [95% CI]			
**Socio-Demographic Factors**				
**Age (years)**	1.17 [1.00–1.36]	1.17 [0.91–1.37]	1.14 [0.97–1.34]	1.22 [0.96–1.54]
**Sex**				
Female	1.00	1.00	1.00	1.00
Male	1.17 [0.69–1.97]	1.92 [0.94–3.90]	1.47 [0.84–2.56]	2.01 [0.90–4.48]
**Place of Residence**				
Beirut (Capital)	1.00	1.00	1.00	1.00
Other Governorates	0.32 [0.10–0.98]	0.21 [0.06–0.71]	0.39 [0.14–1.05]	0.16 [0.04–0.56]
**Father’s Education ^2^**				
Low	1.00	1.00	1.00	1.00
Medium	1.14 [0.57–2.29]	0.83 [0.35–1.96]	0.98 [0.46–2.07]	1.12 [0.43–2.92]
High	0.52 [0.24–1.11]	0.32 [0.11–0.91]	0.73 [0.33–1.62]	0.46 [0.14–1.48]
**Mother’s Education ^2^**				
Low	1.00	1.00	1.00	1.00
Medium	1.98 [0.90–4.33]	1.59 [0.60–4.18]	0.66 [0.30–1.47]	0.68 [0.23–1.95]
High	2.45 [1.13–5.31]	1.36 [0.52–3.59]	0.68 [0.31–1.45]	0.80 [0.29–2.20]
**Mother’s Working Status**				
Not Working	1.00	1.00	1.00	1.00
Working	1.06 [0.55–2.03]	2.60 [1.18–5.70]	2.27 [1.19–4.33]	1.47 [0.60–3.63]
**Crowding Index**				
<1 person/room	1.00	1.00	1.00	1.00
≥1 person/room	1.19 [0.59–2.40]	1.04 [0.40–2.70]	0.64 [0.31–1.31]	0.53 [0.20–1.40]
**Parental Obesity**				
No	1.00	1.00	1.00	1.00
Yes	1.72 [0.97–3.04]	2.67 [1.34–5.31]	2.10 [1.18–3.72]	2.46 [1.15–5.23]
**Lifestyle and Dietary Factors**				
**Physical Activity ^3^**				
Low	1.00	1.00	1.00	1.00
Medium	1.60 [0.91–2.81	1.78 [0.90–3.52]	0.99 [0.52–1.86]	1.43 [0.71–2.89]
High	0.86 [0.56–1.33]	0.75 [0.42–1.33]	0.70 [0.43–1.14	0.68 [0.38–1.22]
**Sedentary Time (h/day)**	1.02 [0.93–1.12]	1.10 [0.97–1.25]	1.05 [0.93–1.19]	1.08 [0.94–1.25]
**Daily Breakfast Consumption**				
No	1.00	1.00	1.00	1.00
Yes	0.20 [0.05–0.84]	0.07 [0.01–0.30]	0.32 [0.07–1.32]	0.25 [0.05–1.25]
**Frequency of Eating Out**				
≤1 time/week	1.00	1.00	1.00	1.00
>1 time/week	1.01 [0.62–1.65]	0.96 [0.48–1.93]	1.18 [0.65–2.14]	1.75 [0.82–3.70]
**Total Daily Energy Intake (kcal) ^2^**				
Low	1.00	1.00	1.00	1.00
Medium	1.21 [0.70–2.10]	1.19 [0.54–2.65]	1.43 [0.73–2.78]	1.13 [0.48–2.66]
High	1.44 [0.71–2.90]	1.10 [0.52–3.74]	1.13 [0.47–2.71]	1.54 [0.54–4.43]
**Bread and Cereals ^4^**				
Low	1.00	1.00	1.00	1.00
Medium	1.19 [0.67–2.12]	0.76 [0.32–1.79]	0.72 [0.35–1.46]	0.51 [0.20–1.32]
High	1.01 [0.53–1.86]	1.36 [0.59–3.14]	0.97 [0.46–2.01]	1.13 [0.46–2.73]
**Milk and Dairies ^4^**				
Low	1.00	1.00	1.00	1.00
Medium	0.92 [0.50–1.69]	0.99 [0.43–2.30]	1.07 [0.52–2.22]	0.53 [0.21–1.32]
High	1.16 [0.62–2.16]	0.64 [0.26–1.56]	0.66 [0.30–1.43]	0.35 [0.13–0.91]
**Meat and Equivalent ^4^**				
Low	1.00	1.00	1.00	1.00
Medium	0.86 [0.48–1.53]	1.19 [0.54–2.61]	1.24 [0.63–2.44]	1.38 [0.56–3.18]
High	0.71 [0.34–1.47]	0.64 [0.21–1.94]	1.32 [0.53–3.29]	1.26 [0.38–4.17]
**Legumes and Nuts^ 4^**				
Low	1.00	1.00	1.00	1.00
Medium	1.08 [0.66–1.76]	0.65 [0.34–1.23]	0.74 [0.41–1.33]	0.86 [0.42–1.74]
High	0.90 [0.51–1.58]	0.68 [0.33–1.42]	0.93 [0.49–1.78]	1.17 [0.55–2.51]
**Fruits and Vegetables ^4^**				
Low	1.00	1.00	1.00	1.00
Medium	1.55 [0.86–2.79]	2.24 [0.99–5.08]	1.08 [0.49–2.37]	2.01 [0.83–4.86]
High	0.91 [0.50–1.67]	1.11 [0.45–2.69]	0.99 [0.48–2.03]	1.11 [0.42–2.90]
**Added Fats and Oils ^4^**				
Low	1.00	1.00	1.00	1.00
Medium	0.73 [0.40–1.32]	0.36 [0.17–0.86]	0.42 [0.20–0.88]	0.79 [0.32–1.93]
High	0.64 [0.34–1.18]	0.30 [0.12–0.73]	0.53 [0.26–1.08]	0.32 [0.12–0.88]
**Fast Foods ^4^**				
Low	1.00	1.00	1.00	1.00
Medium	2.14 [0.91–5.02]	2.41 [0.70–8.23]	1.62 [0.51–5.10]	2.86 [0.64–12.64]
High	3.24 [1.21–8.69]	1.50 [0.43–5.23]	2.46 [0.79–7.67]	1.50 [0.37–6.01]
**Sugar and Sweets ^4^**				
Low	1.00	1.00	1.00	1.00
Medium	1.04 [0.57–1.90]	0.86 [0.37–1.92]	1.03 [0.50–2.14]	1.01 [0.39–2.59]
High	1.12 [0.61–2.06]	0.76 [0.33–1.75]	1.17 [0.57–2.43	1.59 [0.65–3.88]
**Sugar Sweetened Beverages ^4^**				
Low	1.00	1.00	1.00	1.00
Medium	1.13 [0.67–1.90]	0.66 [0.34–1.28]	0.53 [0.22–1.28]	0.57 [0.22–1.17]
High	1.32 [0.79–2.22]	0.59 [0.29–1.17]	0.81 [0.45–1.45]	0.54 [0.20–1.13]

^1^ Overweight and obesity defined based on sex and age specific +1 and +2 BMI *z*-scores, respectively [22]; ^2^ Low, medium and high education levels refer to primary or less, intermediate or high school and above, respectively; ^3^ The three categories of physical activity (Low, Moderate, High) refer to the frequency of physical activity outside the school setting (Never, 1–2 times/week; >2 times/week); ^4^ Food groups’ intake based on percent contribution to daily energy intake. Low, medium, and high refer to first, second, and third tertiles, respectively.

Amongst 12–19-year-old adolescents, male gender was associated with significantly higher odds of obesity (OR = 5.18; 95% CI: 1.76–15.28) and elevated WHtR (OR = 1.82; 95% CI: 1.12–2.97) (Table 5). Similar to findings amongst 6–11-year-old children, significantly lower odds of overweight were observed amongst adolescents residing in other governorates as compared to those living in the capital Beirut (OR = 0.40; 95% CI: 0.19–0.83). Parental obesity was associated with approximately a 3-fold increase in the odds of overweight (OR = 3.01; 95% CI: 1.61–5.63), obesity (OR = 2.93; 95% CI: 1.09–7.86), and elevated WHtR (OR = 2.87; 95% CI: 1.55–5.30). A borderline significant association between high physical activity and lower odds of overweight (OR = 0.62; 95% CI: 0.33–1.05) and central fatness as assessed by WHtR (OR = 0.53; 95% CI: 0.26–1.09) was also observed. Sedentary time was significantly positively associated with all adiposity indicators amongst 12–19-year-old adolescents with higher odds of overweight (OR = 1.12; 95% CI: 1.03–1.21), obesity (OR = 1.2; 95% CI: 1.06–1.35), elevated WHtR (OR = 1.27; 95% CI: 1.13–1.43), and elevated WC (OR = 1.10; 95% CI: 1.01–1.22) being observed. Higher intakes of milk and dairies were associated with significantly lower odds of overweight (OR = 0.56; 95% CI: 0.32–0.98) in this age group. In contrast, higher intakes of sugar sweetened beverages were associated with significantly higher odds of overweight (OR = 2.49; 95% CI: 1.5–4.12) and elevated WHtR (OR = 1.77; 95% CI: 1.02–3.07). A borderline significant association was found between the consumption of fruits and vegetables and lower odds of elevated WC (OR = 0.46; 95% CI: 0.21–1.00) (Table 5).

**Table 5:** Associations of socio-demographic, lifestyle and dietary factors with overweight, obesity, elevated WHtR and elevated WC in Lebanese 12–19-year-old adolescents.

Variables	12–19 Years			
	(*n* = 498)			
	Overweight ^1^	Obesity ^1^	Elevated WHtR	Elevated WC
	Odds ratio [95%CI]			
**Socio–Demographic Factors**				
**Age (years)**	0.99 [0.83–1.18]	0.91 [0.69–1.20]	0.99 [089–1.10]	0.95 [0.77–1.17]
**Sex**				
Female	1.00	1.00	1.00	1.00
Male	1.68 [0.92–3.07]	5.18 [1.76–15.28]	1.82 [1.12–2.97]	0.75 [0.35–1.58]
**Place of Residence**				
Beirut (Capital)	1.00	1.00	1.00	1.00
Other Governorates	0.40 [0.19–0.83]	0.54 [0.14–2.06]	1.18 [0.47–2.96]	1.00 [0.29–3.44]
**Father’s Education ^2^**				
Low	1.00	1.00	1.00	1.00
Medium	1.53 [0.67–3.50]	1.26 [0.30–5.22]	1.70 [0.87–3.31]	1.14 [0.38–3.42]
High	1.47 [0.63–3.42]	2.20 [0.56–8.64]	1.83 [0.90–3.72]	2.13 [0.75–6.08]
**Mother’s Education ^2^**				
Low	1.00	1.00	1.00	1.00
Medium	0.84 [0.33–2.09]	1.33 [0.26–6.86]	0.67 [0.33–1.36]	0.48 [0.15–1.55]
High	1.30 [0.54–3.12]	2.12 [0.50–8.85]	0.73 [0.36–1.45]	0.73 [0.25–2.13]
**Mother’s Working Status**				
Not Working	1.00	1.00	1.00	1.00
Working	1.50 [0.69–3.27]	0.47 [0.10–2.07]	1.10 [0.60–2.02]	1.11 [0.43–2.86]
**Crowding Index**				
<1 person/room	1.00	1.00	1.00	1.00
≥1 person/room	1.74 [0.69–4.40]	0.96 [0.25–3.65]	0.74 [0.39–1.41]	0.73 [0.28–1.95]
**Parental Obesity**				
No	1.00	1.00	1.00	1.00
Yes	3.01 [1.61–5.63]	2.93 [1.09–7.86]	2.87 [1.55–5.30]	1.74 [0.81–3.72]
	**Lifestyle and Dietary Factors**			
**Physical Activity ^3^**				
Low	1.00	1.00	1.00	1.00
Medium	0.79 [0.44–1.39]	0.89 [0.37–2.12]	0.75 [0.39–1.43]	0.77 [0.35–1.69]
High	0.62 [0.33–1.05]	0.43 [0.13–1.33]	0.53 [0.26–1.09]	0.53 [0.21–1.31]
**Sedentary time (h/day) **	1.12 [1.03–1.21]	1.20 [1.06–1.35]	1.27 [1.13–1.43]	1.10 [1.01–1.22]
**Daily Breakfast Consumption**				
No	1.00	1.00	1.00	1.00
Yes	0.62 [0.28–1.40]	1.11 [0.28–4.41]	0.58 [0.24–1.37]	0.72 [0.27–1.88]
**Frequency of Eating Out**				
≤1 time/week	1.00	1.00	1.00	1.00
>1 time/week	0.74 [0.47–1.17]	0.87 [0.42–1.79]	0.87 [0.52–1.45]	1.09 [0.60–1.97]
**Total Daily Energy Intake (Kcal) ^2^**				
Low	1.00	1.00	1.00	1.00
Medium	1.05 [0.56–1.96]	0.62 [0.23–1.69]	0.67 [0.33–1.36]	0.62 [0.28–1.39]
High	0.80 [0.42–1.54]	0.85 [0.31–2.31]	0.75 [0.37–1.52]	0.91 [0.41–2.04]
**Bread and Cereals ^4^**				
Low	1.00	1.00	1.00	1.00
Medium	1.18 [0.69–2.02]	1.71 [0.75–3.92]	1.21 [0.66–2.23]	1.21 [0.61–2.39]
High	0.57 [0.32–1.02]	1.07 [0.43–2.65]	0.79 [0.41–1.50]	0.76 [0.36–1.62]
**Milk and Dairies ^4^**				
Low	1.00	1.00	1.00	1.00
Medium	1.04 [0.61–1.78]	0.79 [0.36–1.75]	1.26 [0.69–2.32]	1.39 [0.70–2.75]
High	0.56 [0.32–0.98]	0.50 [0.21–1.20]	1.17 [0.64–2.15]	1.04 [0.51–2.12]
**Meat and equivalent ^4^**				
Low	1.00	1.00	1.00	1.00
Medium	0.78 [0.44–1.39]	0.89 [0.35–2.27]	0.94 [0.49–1.79]	1.38 [0.64–2.95]
High	1.12 [0.63–1.99]	1.01 [0.39–2.57]	1.01 [0.52–1.93]	1.45 [0.66–3.14]
**Legumes and Nuts ^4^**				
Low	1.00	1.00	1.00	1.00
Medium	0.66 [0.39–1.12]	1.59 [0.76–3.31]	1.34 [0.76–2.46]	1.28 [0.64–2.54]
High	0.97 [0.61–1.53]	0.81 [0.38–1.75]	1.33 [0.77–2.30]	1.25 [0.66–2.36]
**Fruits and Vegetables ^4^**				
Low	1.00	1.00	1.00	1.00
Medium	0.87 [0.50–1.54]	0.73 [0.30–1.73]	0.62 [0.29–1.33]	0.46 [0.21–1.00]
High	0.69 [0.39–1.21]	0.87 [0.36–2.06]	0.61 [0.30–1.24]	0.60 [0.29–1.21]
**Added Fats and Oils ^4^**				
Low	1.00	1.00	1.00	1.00
Medium	1.39 [0.79–2.45]	0.67 [0.28–1.57]	0.87 [0.46–1.65]	0.96 [0.45–2.03]
High	1.19 [0.67–2.13]	0.92 [0.39–2.18]	1.31 [0.70–2.45]	1.63 [0.79–3.35]
**Fast Foods ^4^**				
Low	1.00	1.00	1.00	1.00
Medium	0.92 [0.31–2.73]	0.89 [0.25–1.31]	1.07 [0.44–2.59]	1.64 [0.59–4.57]
High	1.40 [0.53–3.66]	1.34 [0.45–4.03]	1.46 [0.67–3.16]	2.05 [0.83–5.08]
**Sugar and Sweets ^4^**				
Low	1.00	1.00	1.00	1.00
Medium	0.72 [0.41–1.27]	0.82 [0.35–1.88]	0.82 [0.44–1.53]	0.50 [0.25–1.17]
High	0.82 [0.47–1.42]	0.75 [0.32–1.73]	0.82 [0.44–1.53]	0.72 [0.39–1.31]
**Sugar Sweetened Beverages ^4^**				
Low	1.00	1.00	1.00	1.00
Medium	2.49 [1.50–4.12]	1.74 [0.78–3.88]	1.77 [1.02–3.07]	1.62 [0.83–3.13]
High	1.36 [0.80–2.29]	1.69 [0.76–3.77]	0.94 [0.52–1.70]	1.19 [0.60–2.37]

^1^ Overweight and obesity defined based on sex and age specific +1 and +2 BMI *z*-scores, respectively [22]; ^2^ Low, medium and high education levels refer to primary or less, intermediate or high school and above, respectively; ^3^ The three categories of physical activity (Low, Moderate, High) refer to the frequency of physical activity outside the school setting (Never, 1–2 times/week; >2 times/week); ^4^ Food groups’ intake based on percent contribution to daily energy intake. Low, medium and high refer to first, second, and third tertiles, respectively.

## 4. Discussion

Based on a nationally representative survey, this paper reports on the prevalence of overweight, obesity, and abdominal adiposity in Lebanese children and adolescents and provides evidence linking specific dietary, lifestyle, and socioeconomic factors to increased risk of adiposity in this population group. Recognizing that the development of successful obesity prevention strategies should rely on evidence-based public health approaches, the results of this paper could represent a stepping stone for the formulation of effective interventions and policies aiming at curbing the epidemic of obesity in Lebanese youth.

The findings of the present study indicate high prevalence rates of overweight and obesity amongst Lebanese children and adolescents. Using the WHO 2007 BMI criteria, it was found that more than third of 6–19-year-old children and adolescents (34.8%) are overweight (BMI *z* score >+1), with about one in seven (13.2%) being obese (BMI *z* score >+2). To allow comparison with findings reported from selected countries in the region and worldwide, data were re-analyzed according to IOTF and CDC criteria. Based on the IOTF criteria, current prevalence rates of obesity amongst children and adolescents in Lebanon (9.6%) are comparable to those reported from Bahrain (11.3%) [28] and Syria (11.1%) [29], higher than those observed in Qatar (6.3%) [30], while being lower than those reported from the UAE (13.7%) [31]. Based on the CDC 2000 definition, the prevalence of obesity in Lebanese youth (12.6%) appears lower than that reported from the US (18.7%) [32], while being considerably higher than estimates reported from Iran (1.8%) [33] and Saudi-Arabia (5.7%) [34]. When the results of the present study are compared to those provided by the first national survey conducted in 1997 in Lebanon [19,20], an approximate two-fold increase in the prevalence of obesity in 6–19-year-old Lebanese children is noted (7.3% in 1997 *vs.* 13.2% in 2009, based on WHO 2007 criteria). As such, the observed annual increase (+6.7%) in the prevalence of child obesity in Lebanon exceeds the estimated annual increase in the EMRO region (+5.6%), as determined by Wang and Lobstein (2006) [2]. The prevalence of abdominal adiposity in Lebanese youth has followed a parallel increasing trend between 1997 and 2009, with elevated WC rates increasing from 8.5 to 14% and elevated WHtR rates increasing from 19.1 to 21.3% amongst 6–19-year-old children. Current prevalence rates of abdominal obesity as assessed by WHtR (21.3%) were found to exceed those reported from several other countries including Germany (10.7% in boys and 8% in girls) [35] and Pakistan (16.5%) [16] while being lower than those reported from Italy (29.5%) [36]. The prevalence rates of elevated WC (14%) were similar to those reported from Pakistan (13%) [16] and Germany (17.3%) [37], but were lower than estimates reported from Italy (29%) [36].

Gender differentials in the prevalence of obesity and central fatness were noted in 12–19-year-old adolescents, with the odds of obesity being five times higher in boys compared to girls. Adolescent boys were also approximately two times more likely to be abdominally obese compared to girls based on the WHtR indicator. The higher prevalence of obesity amongst boys in this age group, is in line with previous reports from other countries in the region such as Syria, Qatar, Saudi-Arabia, and Greece [29,30,38,39], and with previous studies conducted in Lebanon [19,40]. This may possibly be resulting from stronger cultural and social pressure on adolescent girls to maintain an acceptable body image in this age group [19]. Gender differentials may also be explained by differences in dietary patterns and food choices. In this study, adolescent boys had a significantly higher intake of fast food, sugar sweetened beverages, and breads and cereals, while having significantly lower intakes of fruits and vegetables compared to girls (data not shown).

Our finding of a positive significant association between paediatric adiposity and parental obesity corroborates those reported from other studies and underscores the importance of genetic factors in the aetiology of body fatness [29,41]. However, strong evidence also suggests that childhood obesity is linked to socio-economic development, changes in environmental factors, such as living and school environments, diet, and physical activity patterns [2]. In the present study, specific dietary habits and food choices were associated with the risk of adiposity in the study sample. In 6–11-year-old children, and in line with several previous studies [42,43,44], regular breakfast consumption was associated with a significantly lower risk of overweight and obesity. Although mechanisms linking breakfast consumption to lower body weight are unclear, several possible explanations may exist [42]. Skipping breakfast may lead to excess hunger, rebound overeating [42], and consumption of larger portion sizes [45] and higher amounts of discretionary calories at subsequent meals [42]. Breakfast consumption may also be associated with the selection of more healthful food choices [46], more regular eating habits and increased frequency of eating meals, which is suggested to reduce the efficiency of utilization of metabolizable energy and promote diet-induced thermogenesis and energy expenditure [42,47].

In agreement with previous reports [48,49], fast food consumption was associated with a threefold increase in the risk of overweight amongst 6–11-year-old children. Fast food’s poor nutritional quality [50,51] and higher content of fat and saturated fat [52] underline their potential role as contributors to childhood adiposity and weight gain. Previous studies have shown that compared with non-consumers, children who consume fast food were found to have higher total energy, total fat, and saturated fat intakes [53] and higher obesity risk, while having lower intakes of fiber, milk, fruit, vegetables and fiber [48,49,53,54]. Contrary to the observed association between fast food and adiposity in the study sample, the intake of milk and dairy products was found to be associated with lower odds of abdominal adiposity in this age group (6–11-year-old children). In agreement with our findings, several observational studies have illustrated inverse associations between dairy intake and adiposity in children, while suggesting a role for dairy protein in the regulation of body weight [55,56]. Other studies have found that dietary calcium intake, especially from dairy products, may have a protective effect against overweight and obesity [57,58]. Based on a retrospective analysis of several studies, Heaney *et al.* (2002) proposed that a daily increase of 300 mg of calcium, or approximately 1 dairy serving, was associated with a yearly reduction of approximately 1 kg of body fat in children [59]. It is hypothesized that the relationship between calcium and body weight may be mediated by the lower intracellular calcium levels resulting from high calcium intakes, which reduce lipogenesis while increasing lipolysis and decreasing adiposity [60]. Surprisingly, the intake of “added fats and oils” was found to be associated with a protective effect against obesity and abdominal adiposity in 6–11-year-old children. When looking at the types of fats and oils included in this food group, olive oil was found to contribute 78% of added fats and oils, on a weight basis. Monounsaturated fats (MUFAs) and olive oil, which represent one of the distinctive properties of the Mediterranean diet, was suggested to reduce the risk of obesity in childhood [61]. In a one–year longitudinal study conducted on 13–166-month-old children, the risk of weight gain was significantly lower in children who consumed olive oil compared to those who did not [61]. MUFAs may act on the regulation of appetite, on the intestinal absorption of fat, on the lipolytic activity of the adipocyte and on thermogenesis, among other functions and therefore may contribute to the regulation of body weight [61,62,63,64].

Amongst 12–19-year-old adolescents, and similarly to the findings documented in 6–11-year-old children, higher intakes of milk and dairy products were associated with lower odds of adiposity. In addition, a positive association was documented between higher consumption of sugar-sweetened beverages and a higher risk of overweight and elevated WHtR amongst adolescents. This is in agreement with findings reported from large cross-sectional studies and several well-powered prospective cohort studies [65], which document a positive association between greater intakes of sugar sweetened beverages and obesity in children. A recent meta-analysis of cohort studies found that a higher intake of sugar-sweetened beverages among children was associated with 55% (95% CI 32%–82%) higher risk of being overweight or obese compared to lower intakes [66]. The high added sugar content, low satiety and the resulting incomplete compensation of energy at subsequent meals are likely mechanism by which sugar-sweetened beverages may lead to weight gain [67].

Through combined effects on energy balance, physical activity and sedentary time were suggested as two important and distinct modulators of obesity risk in children and adolescents [68]. In the present study, a borderline significant association was documented between high physical activity and lower odds of overweight and central fatness in adolescents. Similarly, sedentary time was associated with significantly higher odds of overweight, obesity and abdominal adiposity (elevated WC and WHtR) in the same age group. It is suggested that adolescents usually become more interested in screen-time activities such as computer games or watching TV than their younger peers, and, hence, are more prone to engage in sedentary behaviors [69]. When compared to the findings of the previous national survey conducted in 1997 in Lebanon [19], sedentary behavior among Lebanese children and adolescents (defined as ≥10 h sedentary time per day) was found to increase from 19.9% in 1997 to 60.5% in 2009, a finding that may mirror the increased reliance of youth on TV and telecommunication technology. Similarly, regression analyses showed that the risk of overweight/obesity and abdominal obesity was higher in children and adolescents living in the capital Beirut as compared to their counterparts residing in other governorates. Beirut, as a city, is characterized by a complete lack of safe greens and public spaces, such as gardens, parks, playgrounds and sports fields which may have direct repercussions on the lifestyle of children and adolescents such as decreased physical activity, increased screen time and television watching and consequently sedentary behavior [6]. In a European sample of 766 children, aged 10 to 12 years, engagement in more moderate to vigorous physical activity and spending less sedentary time were associated with a more favorable weight status in the study sample [68].

The results of this study document significant associations between certain parental socioeconomic characteristics and adiposity amongst 6–11-year-old children, but not amongst adolescents. An inverse association between fathers’ education level and child obesity was documented. This finding is in disagreement with that reported from several developing countries [29,70] where a positive association between paediatric obesity and higher parental education was documented. However, our findings are in agreement with those reported from developed countries [71,72,73]. A study conducted in Italy among 8- to 9-year-old children showed that the prevalence of paediatric obesity was inversely related to the educational level of fathers, thus highlighting the role of paternal education in modulating the family’s lifestyle, economic and cultural resources, all of which may bear ramifications on nutritional and behavioral choices and therefore obesity risk in childhood [73]. In contrast, and in agreement with findings reported from various developing countries [29,70], higher maternal education was found to be associated with significantly higher odds of overweight amongst 6–11-year-old children. This finding may be a reflection of the association between maternal employment and adiposity in children as the likelihood of employment of the mother increases as her education level increases. In the 6–11-year-old study sample, children with working mothers were found to carry more than a two-fold increase in the risk of obesity and abdominal obesity (elevated WHtR) compared to their counterparts. Maternal employment may in fact be one of the modulators of the family environment, which can have a direct influence on children’s lifestyles, physical activity, and eating habits [74]. A recent longitudinal study in the UK showed that children with working mothers were more likely to be overweight or obese than those of non-working mothers, and children’s likelihood of being overweight or obese increased with the mother’s working time [72].

The results of this study should be considered in light of the following limitations. The use of cut-offs that are not population-specific may jeopardize the sensitivity and specificity of the indices used to assess overweight, obesity and abdominal adiposity. Another limitation of concern is the fact that children aged above 11 years reported themselves on their dietary intake. Children’s recall of food intake may be associated with under-reporting (missing foods), over-reporting [75], as well as incorrect identification of foods due to their lower knowledge of foods and their preparation [76]. It is also important to note that, in our study, dietary information was based on the collection of one 24-h recall, which may not be representative of dietary intakes at the individual level. However, despite its well-known limitations such as reliance on memory and day-to-day variation, the 24-h recall may provide accurate estimates of energy intake at the population level [76]. In the present study, dietary information was collected by the multiple pass 24-h recall approach, which was shown to provide accurate estimates of dietary intake in children [77]. In addition, the recalls were taken by research nutritionists who went through extensive training prior to data collection in order to minimize interviewer errors. Similarly, inter-observer measurement error in anthropometric assessment was minimized by extensive training and follow up to maintain quality of measurement among all research nutritionists. It is important to note that the physical activity questionnaire that was used in this study was not validated. However, the questionnaire was reviewed by a panel of experts including a nutritionist, a physical activity educator and an epidemiologist, and was based on tools used in similar studies.

## 5. Conclusions

This study has documented high prevalence rates of overweight, obesity and adnominal adiposity amongst Lebanese children and adolescents. More importantly, the study’s findings pinpointed towards specific socioeconomic, dietary, and lifestyle factors that may increase the risk of adiposity in Lebanese youth. The documented high prevalence of child adiposity raises questions about its implications for psychosocial development and disease burden in the country, given the association of paediatric adiposity with metabolic syndrome, insulin resistance, hypertension, glucose intolerance, and dyslipidaemia [3,4]. With those below 20 years of age, making up close to 50% of the Lebanese population [78], these estimates do not bode well for the health and well-being of the population. Childhood obesity is related to growing up in an obesogenic environment, in which changes in physical activity and diet appear as the main drivers. In countries undergoing the nutrition transition such as Lebanon, children and adolescents represent the age group that suffers the most from adoption of western lifestyle characterized by long hours of television viewing, computer games, and heavy reliance on fast food, all of which are key factors affecting nutritional habits and obesity levels [79]. In the present study, adiposity in children was positively associated with sedentarity, irregular breakfast consumption, and higher intakes of fast food and sugar-sweetened beverages while the consumption of milk/dairies and olive oil were associated with a lowered risk. Parental socioeconomic characteristics, including education level and maternal employment, were documented as risk factors for adiposity in 6–11-year-old children, but not in adolescents. This highlights the importance of the home environment in modulating the child’s lifestyle and dietary habits and hence obesity risk early in life. Taken together, these findings call for community-based intervention programs that involve multisectoral partnerships and that are responsive to the sociocultural norms of the population. The prevention of paediatric overweight and obesity requires systems-level approaches and environmental support across all sectors of society to achieve sustained dietary and physical-activity behavior change [80]. Based on the results of this study, physical intervention strategies should in particular target adolescents who were shown to have higher levels of sedentarity and to be less likely to engage in physical activity compared to their younger peers. Family-focused interventions and behavioral strategies are needed to instil healthy lifestyle and dietary habits early in life. School-based interventions should integrate behavioral and environmental approaches that focus on dietary intake and physical activity using a systems-level approach [80]. Policy and environmental interventions are recommended as sustainable ways to support healthful lifestyles for children and families and to ensure that all youth have the opportunity to achieve and maintain a weight that is optimal for health [80].
